# Collectanea Medica: Consisting of Anecdotes, Facts, Extracts, Illustrations, &c.

**Published:** 1822-04

**Authors:** 


### COLLECTANEA MEDICA:

CONSISTING OF

### ANECDOTES, FACTS, EXTRACTS, ILLUSTRATIONS, &c.

*Relating to the History or the Art of Medicine, and the Collateral Sciences.*

Floriferis, ut apes, in saltibus omnia libant,
Omnia nos, itidem, depascimur aurea dicta.

---

Art. I.—*Some Experiments made with a View to the Detection and Prevention of Frauds in the Sale of Skimmed Milk; together with an Account of a simple Lactometer for effecting that Purpose.* By Edmund Davy, Esq. Professor of Chemistry, and Secretary to the Royal Cork Institution. (Philosophical Mag. October 1821.)

Skimmed milk, as is well known, is used to a very great extent in Ireland, and especially in the South,* where it forms an indispensable part of the subsistence of the lower orders. Potatoes and skimmed milk, indeed, constitute almost their sole, or at least their principal, food. It is, therefore, of much importance that an article, which essentially contributes to the support of a very large portion of the community, should be supplied in a genuine unadulterated state. Unfortunately, this has not been the case. Much of the skimmed milk exposed for sale in our milk markets, has been largely adulterated with water; and, for want of the means of detection, this fraud has been practised with impunity, not only in Cork, but also in other parts of the country. An unsuccessful attempt has been made to remedy this evil. Persons, called *tasters*, were appointed to inspect the milk markets in Cork, and empowered to detain such milk as they supposed to be adulterated: but the inadequacy of such a mode of prevention must be apparent; for nothing can be more vague and uncertain than decisions founded upon the mere taste and appearance of milk. In consequence of the total incompetency of tasters to prevent the commission of frauds in the sale of skimmed milk, a committee, composed of respectable farmers, &c. was formed for the express purpose of putting a stop to this disgraceful practice, so injurious both to the poor and to the fair dealer. The committee waited upon our active chief magistrate, Sir Anthony Perriere, knt., to apprize him of their intention, and to solicit his assistance. Anxious to co-operate with the committee in promoting a measure of acknowledged public utility, Sir Anthony afforded every assistance in his power, and promised to adopt any practical remedy which should be devised. At his suggestion, the committee consulted me as to the best means of carrying their design into execution; and, in compliance with their request, I directed my best attention to the consideration of the subject. An

---

* The sale of skimmed milk, in the markets of Cork alone, I am informed, amounts to about 1000*l.* per week.

instrument, on the principle of the hydrometer, seemed to promise the simplest means that could be employed for the detection and prevention of frauds in the sale of skimmed milk; but, whether it was practicable to construct such an instrument, depended upon circumstances which could only be determined by experiment. In order to satisfy myself on this point, I commenced a series of experiments on skimmed milk in February last, which have, with occasional interruptions, occupied me ever since. I did not, at first, indulge any sanguine expectations as to the result of my investigation; but, after I had carefully made above a hundred experiments upon *genuine* skimmed milk, procured from many of the principal dairy-farms, embracing all the varieties of cattle, soil, and modes of feeding, common to this part of the country; and also examined many specimens of *adulterated* skimmed milk from the markets, I have at length ventured to construct a simple lactometer, (on the well-known principle of the hydrometer;) the use of which, I have no doubt, will effectually prevent the frauds now practised in the sale of skimmed milk.

Before I describe this instrument, it may be proper briefly to notice the circumstances which led to its construction. My first experiments were directed to ascertain whether any uniformity exists in the density of different specimens of genuine skimmed milk: accordingly, I procured this article from many of the principal dairy-farms, and from private houses in the neighbourhood. I also obtained new milk from the same sources, which I skimmed myself, after suffering the cream to remain on it about the usual time. The greater number of these specimens were of the specific gravity 1.037 and 1.0375. Some were higher; but the highest was 1.040, and the lowest 1.036, the thermometer being at 50°. These experiments, confirmed by others which I afterwards made, led me to conclude that a considerable degree of uniformity prevails in the density of genuine skimmed milk; and this uniformity, I presume, would be still greater, if due allowance were made for accidental circumstances connected with the experiments, which, though not easy to appreciate, must, to a certain extent, influence the specific gravity of milk; as, for example, slight variations of temperature, and of the balance employed; to which must be added the unequal exposure to the atmosphere of the several specimens of milk examined. In reference to this last particular, it is proper to state that I found only one specimen of milk of so high a specific gravity as 1.040; and, in this instance, the cream had been suffered to remain on the milk for above three days, and its specific gravity was not taken until some hours after it had been skimmed. These circumstances incline me to refer its superior density to evaporation, owing to protracted exposure to the atmosphere.

After I was satisfied concerning the degree of uniformity which exists in the density of genuine skimmed milk, my next object was to examine the skimmed milk brought to the markets in Cork, in order to ascertain the nature of the adulterations practised in the sale of this article. Accordingly, I procured, at different times, a great number of specimens from the milk markets; and, on submitting them to a careful examination, I found that some were genuine, and, of course, corresponded with good skimmed milk in every particular. Others were adulterated in different degrees; but the only foreign substance I could detect in the adulterated specimens, was water. By adding a certain quantity of water to genuine skimmed milk, it became of the same density as the adulterated milks from the markets. By simple distillation, the adulterated milks furnished pure water, and became of the same density as genuine milk. In some cases, I found skimmed milk from the markets adulterated with more than one-fifth of water; in other instances, with about one-sixth, one-seventh, and one-eighth of water. The worst of the adulterated milks from the markets was of the specific gravity 1.026; the highest of the genuine milks from the markets was 1.039, the thermometer being at 50°.

It is, I believe, a common opinion that skimmed milk is adulterated with other substances besides water; as, for example, chalk, flour, starch, sugar, &c. which are said to be used for the purpose of concealing the water, by communicating, as circumstances may require, a certain degree of whiteness, thickness, or sweetness to milk. I have made a number of experiments to ascertain the correctness of this notion, and I am convinced the opinion is not well founded. Chalk is perfectly insoluble in skimmed milk, and soon subsides when mixed with it, on account of its superior density. Flour and starch increase the density of skimmed milk; but this effect is only temporary, for, not being soluble, they gradually subside. The high price of sugar, were there no other consideration, precludes its use; for I have found, by experiment, that it would be too expensive, even if it could be procured at the low rate of four-pence per pound.

Those experiments on the density of genuine and adulterated skimmed milk, already noticed, which were made at the temperature of 50°, I have since repeated at 60° Fahr. with similar results, making due allowance for the difference of temperature. I have examined the density of a great number of different specimens of genuine skimmed milk, but have not found any of a lower specific gravity than 1.035 at 60° of Fahrenheit.

All my experiments concur to prove that, in the neighbourhood of Cork, genuine skimmed milk, obtained under nearly similar circumstances, varies comparatively but little in its density; and that the only substance used to adulterate this article for the markets in Cork, is water.

Skimmed milk and water combine without undergoing any sensible alteration of volume, or condensation. Skimmed milk is of much greater specific gravity than water, and its density is diminished in direct proportion to the quantity of water added to it. On those facts, the lactometer I have made depends: it is exclusively adapted to skimmed milk; in which respect, as well as in simplicity of construction, it differs from the ingenious instrument of Mr. Dicas.

### *Description of the Lactometer, &c.*

This instrument differs but little in form from the common hydrometer. Its distinction is to be found in its scale, which is adapted to skimmed milk. It is made of brass, and consists of a pear-shaped bulb, at the top of which is a graduated stem, and at the bottom a brass wire, to the end of which a weight is screwed. The scale begins about three-fourths of an inch from the bottom of the stem, and is marked 0, which corresponds with the specific gravity of the lightest genuine skimmed milk, or 1.035; distilled water being 1.000. The dots and figures, which extend from 0 to 35, indicate "parts of water in 100 parts skimmed milk at 60°," as is engraved on the reverse of the stem, and has been ascertained by experiment. The instrument is constructed for the temperature of 60° of Fahr., a point judged the most convenient, as it agrees, very nearly, with the temperature of the milk brought to our markets during the summer. As all fluids expand by heat and contract by cold, in using the lactometer, an allowance must be made of 1° on the instrument for every 3° of temperature that the milk under examination is either above or below 60° of Fahr. Thus the lactometer, which would remain at 0 in milk of the temperature of 60°, would sink 1° below 0, if the temperature of the milk were increased to 63°; 2°, if it were raised to 66°, &c. And, on the contrary, if the temperature of the same milk were reduced to 75°, the instrument would then experience a rise above 0 equal to 1°, &c. This lactometer is made by Mr. Bennett, mathematical instrument maker, Cork, and sold in a tin case, either with or without a small thermometer. It is scarcely necessary to give directions for using so simple an instrument. All that is required is, to fill the tin case with the milk to be examined, immerse the lactometer in the milk, and observe the point at which it remains stationary after it rises. Note also the temperature of the milk, and, if necessary, make the allowance directed for expansion or contraction of volume.

Before this lactometer was used in the milk markets, experiments were made with it in the presence of the mayor of Cork, Sir A. Perriere, knt., to the committee of whom I have spoken, and other gentlemen, who expressed themselves satisfied with the accuracy and delicacy of the instrument. The first morning it was employed in the milk markets of Cork, the mayor, some of the committee, and myself, attended, when the mayor seized thirty-eight churns of skimmed milk, containing above 2000 pottles. The lactometer stood in most of it at 20°, the thermometer being at 58°, which indicated about one-sixth of water. In the evening of the same day, we again visited the very same markets, but found the milk in all of them so much improved, that not a single churn was seized. Shortly after, a special public meeting of the farmers and dairymen, who supply the markets with milk, was summoned, at which the mayor of Cork presided. In the presence of the meeting he made several experiments with the lactometer, which were deemed satisfactory. The mayor then directed the market jurors, in future, to employ the lactometer in the milk markets, and to detain all skimmed milk in which the instrument sunk 5° below 0, or the point which represents genuine skimmed milk of lowest density. This allowance of 5 was made to avoid being too strict, on the first use of the new instrument. Since the lactometer was first used in the milk markets, the skimmed milk exposed for sale in Cork has been materially improved in quality; and hence comparatively few seizures have been made, though the instrument has now been employed above two months. Dairymen, who have once forfeited their milk, now find they can no longer water it with impunity, and are beginning to relinquish the practice. The same dairy-farms which lately sent milk to the markets in which the lactometer stood at 20°, now furnish milk in which the lactometer stands at 0. Besides the evidence already adduced that water is the only substance used to adulterate skimmed milk in this neighbourhood, the fact has been repeatedly admitted by those concerned in the sale of this article. I have been credibly informed that persons have been hired for the purpose of watering milk, and that in this way hundreds, and even thousands, of pounds have been annually pocketed. This fraud has hitherto been suffered with impunity, merely for want of some simple means of detection. It is every-where so easy and practicable, and may be carried to a great extent without being perceptible to the taste or appearance, though it may be readily discovered by means of the lactometer, which is well adapted, not only for markets where skimmed milk is sold, but also for all public establishments where it is used in large quantities.

---

Art. II.—*Melancholia. Suicide produced solely by a Self-persuasion of its Hereditary Predisposition.* By J. P. Falret. (From a Work in the press, on Mental Alienation.)

A woman, aged thirty-five, of an exceedingly nervous constitution, had laboured, for some time, under symptoms of phthisis pulmonalis, for which complaint she required my assistance. Her childhood had been exempt from any serious malady. Menstruation commenced between fourteen and fifteen years of age, without any unfavourable occurrence. At the age of nineteen, she acquired the knowledge that a paternal uncle had voluntarily put a period to his existence: this discovery very much afflicted her. She had heard it said that insanity was hereditary, and the idea that she might fall into the same unhappy state soon engrossed her whole attention. She carefully concealed from her mother the dismal ideas which continually obtruded themselves on her distempered imagination, but confided them to a clergyman, who made every effort to divert her mind from their influence, though ineffectually. Nevertheless, as her conferences with him procured her some alleviation, she continued them for the space of two years. She was in this melancholy state when her supposed father likewise committed suicide. From that moment, M—— considered herself as inevitably doomed to destruction: she rejected every kind of consolation, and her approaching end entirely pervaded her imagination. A thousand times she was heard to say, "I am doomed, then, to perish like my father and uncle: my blood is contaminated." This last thought acquired the highest degree of certainty in her mind, when, at the period of menstruation, which soon after happened, she noticed the discharge was both less in quantity and of a much lighter colour.

She no longer doubted but that her blood was entirely decomposed; and, driven to desperation, she formed the resolution of drowning herself: to accomplish which, (after having left in her mother's chamber a letter, informing her of the fate of her unfortunate child,) she precipitated herself into the river, from whence, however, she was extricated, and restored to life.

She passed the following night in great agitation. Intolerable pains, particularly in the frontal region, prevented sleep before one the following morning. Upon awaking two hours after, she no longer recognized the place of her abode or any of her attendants: she was in a state of delirium, but uttered nothing in allusion to her former melancholy condition. It is worthy of notice, that this unfortunate girl, who was previously very reserved in her expressions, and had paid much attention to her religious duties, seemed now delighted by the utterance of the most obscene expressions. This maniacal delirium, which continued three days, was followed by melancholy, accompanied by a powerful inclination to suicide.

Cephalitis re-appeared, but with less intensity. M—— likewise experienced a slight nausea, accompanied by vomiting of yellow matter, which soon subsided. Her natural *embonpoint* sensibly diminished. In a very short time, the menstrual evacuation became irregular, and returned about every twenty days. Gloomy despair was depicted on her countenance; she could not see herself in a glass without experiencing a sensation of terror, (according to her own expression.)

Such was the state in which she laboured, when the aid of religion, a second time, partially alleviated her sufferings; but which was insufficient, entirely, to relieve her apprehensions. At this period, her mother endeavoured to procure an interview with her real father. After many unsuccessful attempts, a day was fixed. Though she could not, at first, be convinced of the truth of the narrative, she consented to see the author of her existence; and the physical resemblance between them instantly dispelled her suspicions. From that time M—— banished all thoughts of self-destruction: her natural gaiety returned, and she regained her health. Menstruation, alone, maintained its irregularity for the three ensuing months.

Fourteen years have elapsed since she attempted suicide. M——, during this interval, has become the mother of three children; and, although, since her marriage, she has sustained more misfortunes (amongst others, poverty, having been forced to obtain succour from charitable institutions,) than during the time she lived with her mother, there has never since appeared the slightest inclination to renew her former horrid attempt. She enjoys the free exercise of all her intellectual faculties: and, I understand, her children receive from her the most tender cares and affections.

This case, which bears the greatest analogy to that published by Dr. Villermé, in the Bulletin of August, presented to the Medical Society of Emulation, confirms many general ideas contained in that memoir.

With regard to the cause, this melancholy may be considered as similar to that which developes itself sometimes at the commencement of intermittent mania. Still retaining sufficient faculties to judge of the imminence of a disease so horrible, these patients are then in a state the more dreadful, as the recollection of former paroxysms are presented to their minds with more force than under any other circumstances. The afflictions of the present, with the painful memory of the past, are also accompanied by a dread of the future, which renders this state one of the most terrible that can be imagined.

What situation, in fact, can be more lamentable than that of a man who has the total annihilation of his reason constantly presented to his distempered imagination?

Was not this the case, in a certain degree, with the subject of this article? She is convinced of possessing an hereditary predisposition to suicide: is it to be wondered at, that the influence of the idea should impel her to the commission of it? If the maniac has the experience of his former paroxysm, as a basis of his determination, cannot she, likewise, urge the example of her father and uncle?

This fact is an additional proof of the original disorganization of the brain in mental alienation: the cause productive of the disease, its course, and the method of cure, cannot leave the slightest doubt of it. The phenomena exhibited by the stomach and uterus are evidently sympathetic.

The presence of delirium, both antecedent to and after the attempt at suicide, is a circumstance which strongly militates against those authors who consider it as an act committed in cold blood.

The depravations of the intellectual faculties appeared under two forms, viz. melancholic delirium, and a maniacal one, which lasted but three days. Regarded in this point, that fact must be added to those adduced to prove that the importance of the form of delirium has been exaggerated in the treatment of mania.

I could make many more remarks on this case, but they will naturally present themselves to every reader. I shall terminate by one reflection, which may be of some interest at an epoch when several medical men, of well-known knowledge and philanthropy, propose to renew the laws enacted against *felo de se.*

We may, in a certain degree, conceal from the children that a suicide has been committed, at any past period, in the family; but, if it gains publicity by the enforcement of a rigorous law, these children must inevitably become acquainted with it; and this dreadful circumstance will merely serve to increase, as is attested by the preceding narrative, the unhappy *predisposition.* This word suggests to me an objection, which I consider as a strong one, against the medical men above mentioned. What? is it acknowledged that suicide is hereditary, and yet do we invoke the severity of the laws to punish it? It is, then, wished that society, in general, should hasten to mark its victim even in the breast of its parent?

---

Art. III.—*Singular Case of Lithotomy.* (From the Revue Medicale for December 1821.)

Dr. Soubervielle has presented, to the Medical Society of Emulation, a patient, named Dapret, thirty years of age, a private of the 135th regiment of the line.

This man was wounded by a gun-shot, in the inferior margin of the false ribs of the left side, at the engagement near Halle, on the 2d of May, 1813, and recovered in a short time, having had several fragments of his garments extracted from the wound. The ball, by which it was presumed to have been inflicted, could not be discovered.

In 1815, a tumor, accompanied by fever and other inflammatory symptoms, appeared in the hypogastric region of the wounded side. About six weeks after, the patient experienced a sensation of pain, answering to that felt by the laceration of any internal part, towards the bladder, which was followed by a pressing desire to make water: the tumor, at the same time, became much depressed. He voided a large quantity of urine, mixed with pus and blood. After a few days, there was no appearance of the latter, but his water continued purulent for some time.

From this period, the patient experienced a constant pain in the region of the bladder, and passed more or less blood, according to the degree of exercise his occupations required. He was examined by a skilful surgeon of Paris, who thought he could feel some extraneous body in the bladder; but, at the moment every thing was in readiness to remove it by an operation, the substance could not be felt.

Several surgeons, afterwards, introduced the sound at different periods, as the pain still continued unabated, without finding any stone. Dr. Soubervielle having been consulted, convinced himself of its presence, and, on the 23d of August, proceeded to remove it by the lateral operation. He succeeded in extracting a stone equal in size to a hen's egg, the nucleus of which was found to be a grape-shot—one-half of which, only, was incrusted with calcareous matter. The patient completely recovered in eighteen days.

This case is particularly interesting, from various circumstances; for, though calculi have been extracted from the bladder, the nuclei of none were found equal in magnitude to a grape-shot. The nucleus being, likewise, but half covered, it is to be presumed that the plain side could not come in contact with the urine. To support this assertion, it is to be remarked, that the calcareous matter becomes gradual towards the circumference, where it terminates: a number of streaked lines are also visible on the uncovered side of the shot, in a situation perpendicular to the circular termination of the calcareous covering.

Several questions naturally suggest themselves to the reader in this case; but Dr. Soubervielle considers it his duty merely to relate facts. Every one may interpret, in his own way, whatever circumstances may not be submitted here.

---

Art. IV.—*On Irritation of the Spinal Nerves.* By Mr. Richard P. Player. (From the Journal of Science, No. xxiv.)

I take the liberty to submit to your notice a pathological fact, which has not, to the best of my knowledge, been generally remarked, and attention to which, as far as my own experience goes, promises some diminution of those difficulties with which the healing art has to contend. Most medical practitioners, who have attended to the subject of spinal disease, must have observed that its symptoms frequently resemble various and dissimilar maladies, and that, commonly, the function of every organ is impaired, whose nerves originate near the seat of the disorder. The occurrence of pain in *distant parts* forcibly attracted my observation, and induced frequent examinations of the spinal column; and, after some years' attention, I consider myself enabled to state, that, in a great number of diseases, morbid symptoms may be discovered about the origins of the nerves which proceed to the affected parts, or of those spinal branches which unite with them; and that, if the spine be examined, more or less pain will commonly be felt by the patient, on the application of pressure about or between those vertebræ from which such nerves emerge. If disease be confined to one side of the body, or one arm or one leg, this tenderness will be felt on the *same side* of the spine *only;* but, if central parts, or both sides of the body, or both arms or legs, are diseased, tenderness will be felt on *both sides* of the spine. This symptom has been found to attend various other affections. This spinal affection may, perhaps, be considered as the *consequence* of diseases, but of its existence at their commencement any person may satisfy himself; and this circumstance, combined with the success which has attended the employment of topical applications to the tender parts about the vertebræ, appear to indicate that the cause may exist there. Prejudice sometimes operates against the idea of connexions so remote; but, in many instances, patients are surprised at the discovery of tenderness in a part, of whose implication in disease they had not the least suspicion.

The opinion entertained by some of our continental neighbours of the importance of the spinal brain in disease, is well known. That many of our maladies are the sympathetic consequences of the operation of a distant cause, and that diseases, apparently the most dissimilar, may have one common origin, have been the doctrines of some of our own most eminent pathologists; and, though the injuries inflicted on our frames by mechanical and chemical agents usually manifest their effects at the part where the cause has acted, we may be too much disposed to generalize from these premises, and to conclude that the cause of pain, inflammation, and the other phenomena of disease, also exist at the part where the symptoms are perceived. The records, however, of disease abound with cases, which, combined with daily observation and the discoveries of morbid anatomy, tend to shake our confidence in this very natural and general inference, and to indicate that the cause can, and frequently does, exist very remotely from its phenomena; and that vascular fullness about the origins of nerves can produce the most formidable symptoms in the parts to which they are distributed. Such instances I need not enumerate; but may be permitted to remark, that they appear to corroborate the opinion that all the degrees of morbid sensation, from the slightest tingling and itching to the severest pain, may be produced by one common cause, obstructed nervous function, of which the ultimate effects are spasm, convulsion, and paralysis.

